# Effect of feeding rice based distillers dried grains with solubles and gluten meal on nutrient transporter genes and immunity in broiler chickens

**DOI:** 10.14202/vetworld.2018.1592-1596

**Published:** 2018-11-20

**Authors:** Om Prakash Dinani, Pramod Kumar Tyagi, Asit Baran Mandal, Praveen Kumar Tyagi, Dukare Sagar Popat, Sita Prasad Tiwari

**Affiliations:** 1 Division of Avian Nutrition and Feed Technology, ICAR-Central Avian Research Institute, Bareilly, Uttar Pradesh, India; 2 Department of Animal Nutrition, College of Veterinary Science and Animal Husbandry, Durg, Chhattisgarh, India

**Keywords:** broiler, immunity, nutrient transporter genes, protease, rice distillers dried grains with solubles, rice gluten meal

## Abstract

**Aim:**

The aim of this study was to investigate the effect of feeding rice based distillers dried grains with solubles (rDDGS) and gluten meal on nutrient transporter genes and immunity in broiler chickens.

**Materials and Methods:**

A 2×3 factorial design resulted in six experimental diets, namely T1 (no rDDGS/rice gluten meal [RGM]/enzyme), T2 (no rDDGS/RGM, with multienzymes), T3 (12.5% rDDGS, 15% RGM, no enzyme), T4 (12.5% rDDGS, 15% RGM, with protease enzyme), T5 (10% rDDGS, 12.5% RGM, no enzyme), and T6 (10% rDDGS, 12.5% RGM, with protease enzyme). Each treatment was allocated five replicates of chicks, with eight birds in each. Nutrient transporter genes such as Mucin (MUC 2), excitatory amino acid transporter 3 (EAAT3), and peptide transporter (PepT1) and immunity were estimated using standard procedures.

**Results:**

Feeding rDDGS and RGM combination improved humoral immunity, while cell-mediated immunity did not show any significant (p>0.05) effect on broiler chickens. MUC and PepT1 genes showed significantly (p<0.01) decreased relative fold expression in 12.5% rDDGS +15% RGM combination, while EAAT3 gene showed significantly (p<0.01) decreased relative fold expression in both rDDGS and RGM combination levels.

**Conclusion:**

Thus, it may be concluded that feeding rDDGS and RGM combination improved humoral immunity but had an adverse effect on nutrient transporter gene in broiler chickens.

## Introduction

Poultry industry is the fastest growing sector in Indian agriculture. Feed is the major constituent in the poultry production accounting for 65-75% of total recurring expenditure. Feed costs are primarily driven by the cost of protein sources. Substitution of expensive protein sources with lower cost ingredients would potentially reduce the cost of the feed [1].

The rice production in our country was about 106.65 MT in 2013-2014, and rice tops the list of total cereal production in the country [2]. Nowadays, certain newer rice by-products are available in appreciable quantities, and cheaper rate that can be utilized as protein sources from rice processing industries such as rice distillers dried grain with solubles (rDDGS) and rice gluten meal (RGM). DDGS is a coproduct of the ethanol industry produced during the dry milling process. Its availability is increasing due to higher demand for ethanol as biofuel [3].

Very few researches were done in rDDGS and gluten meal regarding immunity and gene expression [4-6] in broiler. No study is available in the literature regarding feeding of rDDGS and RGM in combination. Hence, there is a need to explore regarding effect of feeding rDDGS and gluten meal replacing costly soybean meal (SBM) in broiler diet on immunity and gene expression.

## Materials and Methods

### Ethical approval

All the procedures carried out on the birds were approved by the Institutional Animal Ethics Committee (IAEC) and Committee for the Purpose of Control and Supervision of Experiments on Animals (CPCSEA) of ICAR-Central Avian Research Institute, Izatnagar – 243 122, Uttar Pradesh. The IAEC/CPCSEA number is 452/01/ab/CPCSEA.

### Experimental design and diets

A total of 240 CARIBRO-Vishal broiler chicks of the same hatch and uniform weight were procured from the institutional hatchery and housed in specially designed battery brooder cages with standard watering and feeding facilities. The broiler chicken ration was formulated by recommendation of the ICAR [1] using rDDGS and RGM as replacement of SBM in the basal diets along with enzyme supplementation. The levels of rDDGS, RGM, and the suitable enzymes were standardized in a preliminary trial. The rDDGS levels of 10% and 12.5% and RGM levels of 12.5% and 15% along with either multienzymes or protease enzyme were selected to formulate the experimental diets as the pre-starter, starter, and finisher diets. The feed ingredients and the nutrient composition of diets have been given in Table-1.

**Table-1:** Ingredient and nutrient composition of broiler chicken diets.

Diet composition	Pre-starter diet (0-14 days)						Starter diet (14-28 days)						Finisher diet (28-42 days)					
		
	T1	T2	T3	T4	T5	T6	T1	T2	T3	T4	T5	T6	T1	T2	T3	T4	T5	T6
Ingredients																		
Maize	54.42	54.42	58.58	58.58	57.78	57.78	55.63	55.63	60.97	60.97	59.81	59.81	62.00	62.00	66.77	66.77	65.61	65.61
SBM	38.40	38.40	8.80	8.80	14.30	14.30	37.10	37.10	7.40	7.40	13.00	13.00	31.30	31.30	2.00	2.00	7.50	7.50
DORB	0.00	0.00	0.70	0.70	0.90	0.90	0.00	0.00	12.50	12.50	10.00	10.00	3.22	3.22	0.00	0.00	0.70	0.70
DDGS	0.00	0.00	12.50	12.50	10.00	10.00	0.00	0.00	15.00	15.00	12.50	12.50	0.00	0.00	12.50	12.50	10.00	10.00
RGM	0.00	0.00	15.00	15.00	12.50	12.50	3.50	3.50	0.20	0.20	0.80	0.80	0.00	0.00	15.00	15.00	12.50	12.50
Oil	3.00	3.00	0.00	0.00	0.40	0.40	1.35	1.35	1.15	1.15	1.23	1.23	0.50	0.50	0.50	0.50	0.50	0.50
LSP	1.40	1.40	1.30	1.30	1.10	1.10	1.55	1.55	1.70	1.70	1.67	1.67	0.70	0.70	0.40	0.40	0.33	0.33
DCP	1.82	1.82	2.00	2.00	2.00	2.00	0.00	0.00	0.32	0.32	0.22	0.22	1.45	1.45	1.70	1.70	1.64	1.64
Lysine	0.00	0.00	0.35	0.35	0.25	0.25	0.10	0.10	0.00	0.00	0.00	0.00	0.00	0.00	0.36	0.36	0.30	0.30
Methionine	0.20	0.20	0.00	0.00	0.00	0.00	0.765	0.765	0.765	0.765	0.765	0.765	0.06	0.06	0.00	0.00	0.00	0.00
Constant*	0.765	0.765	0.765	0.765	0.765	0.765	55.63	55.63	60.97	60.97	59.81	59.81	0.77	0.77	0.77	0.77	0.77	0.77
Enzyme	-	M	-	P	-	P	-	M	-	P	-	P	-	M	-	P	-	P
Nutrient composition																		
CP (%)	21.99	21.99	22.02	22.02	22.02	22.02	21.52	21.52	21.50	21.50	21.49	21.49	19.51	19.51	19.52	19.52	19.49	19.49
ME (kcal/kg)	2998	2998	3002	3002	2998	2998	3050	3050	3051	3051	3050	3050	3100	3100	3099	3099	3104	3104
Ca (%)	1.03	1.03	1.08	1.08	1.00	1.00	0.95	0.95	0.95	0.95	0.96	0.96	0.86	0.86	0.85	0.85	0.86	0.86
Available P (%)	0.45	0.45	0.45	0.45	0.46	0.46	0.41	0.41	0.40	0.40	0.40	0.40	0.38	0.38	0.39	0.39	0.39	0.39
Lysine (%)	1.19	1.19	1.19	1.19	1.20	1.20	1.38	1.38	1.12	1.12	1.13	1.13	1.20	1.20	1.00	1.00	1.04	1.04
Methionine (%)	0.52	0.52	0.53	0.53	0.51	0.51	0.48	0.48	0.53	0.53	0.50	0.50	0.41	0.41	0.50	0.50	0.48	0.48
Threonine (%)	0.80	0.80	0.81	0.81	0.82	0.82	0.79	0.79	0.81	0.81	0.80	0.80	0.86	0.86	0.79	0.79	0.81	0.81
Cost (Rs./kg)	28.52	28.93	23.02	23.63	23.68	24.29	28.03	28.43	22.88	23.48	23.65	24.25	26.72	26.72	22.03	22.03	22.93	22.93

SBM=Soybean meal, DORB=De-oiled rice bran, DDGS=Dried distillers grains with solubles, RGM=Rice gluten meal, LSP=Limestone powder, DCP=Dicalcium phosphate, CP=Crude protein, ME=Metabolizable energy, M=Multienzymes, P=Protease.

* Constant (0.4% salt, 0.1% trace mineral premix, 0.15% vitamin premix, 0.015% Vitamin B complex, 0.05% choline chloride, and 0.05% Toxin binder). 1 - Trace mineral premix supplied (mg/kg diet): Mg 300; Mn 55; I 0.4; Fe 56; Zn 30; Cu 4. 2 - Vitamin premix supplied (per kg diet): Vitamin A 8250 IU; Vitamin D3 1200 IU; Vitamin K 1mg; Vitamin E 40 IU. 3 - B complex: Vitamin B1 2 mg; Vitamin B2 4 mg; Vitamin B12 10 µg; niacin 60 mg; pantothenic acid 10 mg; choline 500 mg

A 2×3 factorial design resulted in six experimental diets, namely T1 (no rDDGS/RGM/enzyme), T2 (no rDDGS/RGM, with multienzymes), T3 (12.5% rDDGS, 15% RGM, no enzyme), T4 (12.5% rDDGS, 15% RGM, with protease enzyme), T5 (10% rDDGS, 12.5% RGM, no enzyme), and T6 (10% rDDGS, 12.5% RGM, with protease enzyme). Each treatment was allocated five replicates of chicks, with eight birds in each. The feeding trial was conducted for 6 weeks, and the feed, as well as drinking water, was provided *ad libitum* to the birds during the entire experimental period. The biological experiment was conducted from February 20 to April 3, 2018, recorded mean poultry shed temperature of 28±0.57–32±0.84°C and relative humidity of 52±0.80–57±0.63%. Continuous lighting was provided for 23 h at the intensity of 0.72±0.05–0.85±0.02 foot-candle at bird eye level in battery cages. Stocking density was maintained in terms of eight birds per battery cage with standard floor space.

### Immunological parameters

The antibody response to sheep red blood cells was determined using a standard hemagglutination assay for humoral immunity [7,8]. The *in vivo* cell-mediated immune (CMI) response to phytohemagglutinin type P was evaluated by the method of Cheng and Lamont [9]. Eight birds (4 male and 4 female) from each treatment were selected for assessing cell-mediated immunity (CMI) and humoral immunity.

### Genomic expression

Four experimental birds (2 male and 2 female) from each treatment were sacrificed at the 21^st^ day of experiment for the expression of gut-related genes such as mucin (MUC 2), excitatory amino acid transporter 3 (EAAT3), and peptide transporter (PepT1). The jejunum tissues were collected aseptically and preserved for RNA estimation. The birds were slaughtered humanely at the Division of Avian Nutrition and Feed Technology, ICAR-CARI, Izatnagar. Isolation of total RNA from tissue samples was done by an established TRIzol method in our laboratory. The PCR conditions followed for cDNA synthesis will be as: 25°C for 5 min, 42°C for 60 min, and 70°C for 5 min. The resultant first strand cDNA was stored at −80°C until further analysis. Annealing temperature used for this study was standardized in our laboratory. The annealing temperature for PepT1, EAAT3, MUC, and GAPDH genes was 51, 57, 58, and 56°C, respectively.

### Statistical analysis

Data subjected to test of significance as per 2×3 factorial completely randomized design were analyzed for mean, standard errors, and analysis of variance using Statistical Package for the Social Sciences (SPSS) 16.0 version, and comparison of means was done using standard procedures [10,11]. The gene expression data were analyzed by software REST 2009 version (Developed by Technical University, Munich and QIAGEN company, Germany).

## Results

### Immunological parameters

The data pertaining to feeding different levels of rDDGS and RGM combinations with or without enzymes on immunological parameters in terms of humoral and CMI are presented in Table-2. The results revealed that 0% rDDGS +0% RGM (DR1), 12.5% rDDGS +15% RGM (DR2), and 10% rDDGS +12.5% RGM (DR3) combination levels did not show any significant (p>0.05) difference on CMI. However, humoral immunity was improved significantly (p<0.05) in DR2 and DR3 as compared to DR1. There was no significant (p>0.05) difference in humoral immunity in DR2 as compared to DR1. Effect of with or without enzymes and their interaction of rDDGS and RGM combinations did not show any significant (p>0.05) difference on humoral and CMI.

**Table-2:** Effect of feeding different levels of rDDGS and RGM combinations on immunological parameters.

Treatment	rDDGS%	RGM%	Enzyme	HA (log2)	CMI (mm)
T1	0	0	-	2.71	55.75
T2	0	0	M	2.75	57.00
T3	12.5	15	-	2.87	57.88
T4	12.5	15	P	2.90	60.38
T5	10	12.5	-	2.85	58.25
T6	10	12.5	P	2.85	58.75
			Pooled SEM	0.02	0.48
		rDDGS%	RGM%		
		0	0	2.72^a^	56.37
		12.5	15	2.88^b^	59.12
		10	12.5	2.84^b^	58.50
			Enzyme		
			Without	2.81	57.29
			With	2.83	58.71
			Significance		
			rDDGS+RGM	p<0.05	NS
			Enzyme	NS	NS
			Interaction	NS	NS

Values bearing different superscripts within the column differ significantly. NS=Non-significant (p>0.05), M=Multienzymes, P-Protease, rDDGS=Rice based distillers dried grains with solubles, RGM=Rice gluten meal

### Gene expression

The results pertaining to feeding different levels of rDDGS and RGM combinations with or without enzymes on gene expression (relative fold expression) are presented in Table-3. MUC 2, EAAT3, and PepT1 genes related to nutrient transport were studied for this experiment on 21 days taking jejunum tissue. The results revealed that 0% rDDGS +0% RGM (DR1), 12.5% rDDGS +15% RGM (DR2), and 10% rDDGS +12.5% RGM (DR3) combination levels showed that MUC and PepT1 genes showed significantly (p<0.01) decreased relative fold expression in DR2 level as compared to DR1 and DR3 levels. Relative fold expression of MUC and PepT1 genes did not show any significant (p>0.05) difference in DR3 as compared to DR1 level. EAAT3 gene showed significantly (p<0.01) decreased relative fold expression in DR2 and DR3 levels as compared to DR1 level. Relative fold expression of EAAT3 genes did not show any significant (p>0.05) difference in DR3 as compared to DR2 level. Effect of with or without enzymes and their interaction with rDDGS and RGM combinations did not show any significant (p>0.05) difference on MUC, EAAT3, and PepT1 gene expression.

**Table-3:** Effect of feeding different level of rDDGS and RGM combinations on gene expression (relative fold expression).

Treatment	rDDGS%	RGM%	Enzyme	MUC	EAAT	PEP
T1	0	0	-	1.00	1.02	0.99
T2	0	0	M	1.03	1.03	0.91
T3	12.5	15	-	0.86	0.87	0.71
T4	12.5	15	P	0.87	0.91	0.71
T5	10	12.5	-	1.00	1.00	0.77
T6	10	12.5	P	1.05	0.99	0.77
			Pooled SEM	0.02	0.02	0.03
		rDDGS%	RGM%			
		0	0	1.01^b^	1.02^b^	0.95^b^
		12.5	15	0.86^a^	0.89^a^	0.71^a^
		10	12.5	1.02^b^	0.99^a^	0.77^b^
			Enzyme			
			Without	0.95	0.96	0.82
			With	0.98	0.98	0.79
			Significance			
			rDDGS+RGM	p<0.01	p<0.01	p<0.01
			Enzyme	NS	NS	NS
			Interaction	NS	NS	NS

Values bearing different superscripts within the column differ significantly. NS=Non-significant (p>0.05), M=Multienzymes, P=Protease, rDDGS=Rice based distillers dried grains with solubles, RGM=Rice gluten meal, EAAT=Excitatory amino acid transporter 3, MUC=Mucin

## Discussion

Our results are in agreement with researchers Barekataina *et al*. [12] and Wani [6] but disagreement with Gupta [5] in terms of immune response as result of feeding rDDGS and gluten meal. Sorghum DDGS at 20% level with or without combination of protease and xylanase in broiler chickens improved (p<0.01) the IgA and IgG titer at day 13 under disease condition [12]. No significant (p>0.05) difference was found on dietary inclusion of RGM up to 20% level with or without protease enzyme and their interaction on humoral immunity and CMI [6]. Contrary to this, immune response was found non-significant (p>0.05) when rDDGS was included in the diets at varying levels (5%, 7.5%, and 10%) with or without protease supplementation [5]. Better humoral immunity in our experiment may be associated with a higher level of methionine amino acid in rDDGS and RGM-based diets.

Our results are in agreement with Gilbert *et al*. [4] and Wani [6] in terms of gene expression as a result of feeding rDDGS and gluten meal. The PepT1 and EATT3 were influenced by dietary protein quality as well as feed restriction, with greater expression in chicks that fed high-quality protein SBM compared with chicks that consumed corn gluten meal [4]. Downregulation in PepT1 and MUC gene at 21 days post-hatch and EAAT3 gene expression did not reveal any significant (p>0.05) difference in 20% RGM with protease group compared to control in broiler chickens [6]. No other reference is available regarding the effect of rDDGS and RGM combination on gene expression. Downregulation in nutrient transporter genes in our experiment may be associated with poor digestibility of rDDGS and RGM protein sources as compared to SBM.

## Conclusion

It may be concluded that feeding rDDGS and RGM combination improved humoral immunity, while CMI did not show any significant effect. MUC and PepT1 genes showed significantly (p<0.01) decreased relative fold expression in 12.5% rDDGS +15% RGM combination, while EAAT3 gene showed significantly (p<0.01) decreased relative fold expression in both rDDGS and RGM combination levels.

## Authors’ Contributions

PramodKT conceived the work. ABM designed the experiments. OPD collected samples and performed the experiments. PKT performed the statistical analysis. DSP helped during experiments. SPT guided to draft the manuscript. All authors read and approved the final manuscript.
